# The influencing factors of amplitude-integrated electroencephalography and bilirubin-induced neurological dysfunction scores in neonates with hyperbilirubinemia: a cross-sectional study

**DOI:** 10.1186/s12887-026-06700-1

**Published:** 2026-03-06

**Authors:** Jie Chen, Linglin Xia, Jiaojiao Wu, Wenjie Dai, Yitong Zhang, Kerong Li, Yan Ma

**Affiliations:** The Affiliated Yan’an Hospital of Kunming Medical University, Kunming, China

**Keywords:** Amplitude integrated electroencephalogram (aEEG), Bilirubin-Induced Neurological Dysfunction (BIND), Hyperbilirubinemia, Bilirubin-induced brain injury, Neonates

## Abstract

**Background:**

Amplitude-integrated electroencephalography (aEEG) and bilirubin-induced neurological dysfunction (BIND) scores are important bedside tools for evaluating neonatal brain function, particularly regarding bilirubin-induced brain injury. This study aimed to analyze the influencing factors of aEEG and BIND scores in neonates with hyperbilirubinemia.

**Methods:**

A total of 294 neonates with gestational ages of ≥ 35 weeks, diagnosed with hyperbilirubinemia were enrolled. All infants underwent bedside aEEG monitoring and BIND scoring within 48 h after admission, with assessors blinded to laboratory results. Clinical and laboratory data were collected. Univariate analysis, logistic regression, correlation analysis, and receiver operating characteristic (ROC) curve analysis were performed.

**Results:**

① The indirect bilirubin/albumin ratio (IB/A) was significantly associated with abnormal aEEG scores (OR = 1.722, 95% CI = 1.436–2.066) and abnormal BIND scores (OR = 1.251, 95% CI: 1.050–1.491). Sensitivity analysis confirmed similar associations for total bilirubin (TB), indirect bilirubin (IB), and total bilirubin/albumin ratio (TB/A). ② The area under the ROC curve (AUC) of IB/A for identifying abnormal aEEG and abnormal BIND scores was 0.736 and 0.587, respectively. The optimal cutoff values (8.7 and 7.7) showed high specificity (95.2% and 70.5%) but low sensitivity (43.9% and 47.1%). ③ aEEG scores were negatively correlated with TB, IB, TB/A, IB/A, albumin (ALB), and corrected gestational age (CA) (all *P* < 0.05). BIND scores were negatively correlated with aEEG scores and positively correlated with TB/A (both *P* < 0.05). Birth weight, Birth length, maternal age, day age, and lactate dehydrogenase (LDH) showed no significant correlations with either score (all *P* > 0.05).

**Conclusions:**

In neonates ≥ 35 weeks of gestation with hyperbilirubinemia and mainly mild neurological dysfunction, serum bilirubin burden represented by IB/A is an independent core factor associated with abnormal bedside aEEG and BIND scores. Conventional demographic and perinatal indexes, including birth weight, birth length, maternal age, day age, and delivery mode, show no significant influence on the two scores in this study population.

**Supplementary Information:**

The online version contains supplementary material available at 10.1186/s12887-026-06700-1.

## Background

Neonatal hyperbilirubinemia is one of the most common conditions encountered in the neonatal period, with etiologies ranging from physiological jaundice and breastfeeding-associated jaundice to pathological forms induced by hemolytic diseases (e.g., Rh/ABO incompatibility, glucose-6-phosphate dehydrogenase [G6PD] deficiency), infections, and hepatobiliary anomalies. When serum bilirubin levels are abnormally elevated, especially when the level of unbound unconjugated bilirubin (UB) is excessively high, it may cross the immature blood-brain barrier of neonates, triggering acute bilirubin encephalopathy (ABE) or even chronic kernicterus, resulting in permanent neurological sequelae such as auditory impairment, motor disorders, and cognitive deficits [1]. While phototherapy and exchange transfusion are well-established effective interventions for preventing and managing severe hyperbilirubinemia and ABE, the timing and intensity of these treatments hinge critically on the early and accurate assessment of bilirubin neurotoxicity risk [2]. Clinically, infants with ABE may present with concurrent conditions such as infection, acidosis, hypoglycemia, and hemolysis, among others. These comorbidities may affect the permeability of the blood-brain barrier or exacerbate the neurotoxicity of bilirubin, further complicating early identification and assessment [3–5].

The diagnosis of bilirubin encephalopathy primarily depends on magnetic resonance imaging (MRI) and auditory brainstem response (ABR). However, the sensitivity and specificity of MRI require improvement, as its abnormalities in mild-to-moderate hyperbilirubinemia are often overlooked. Furthermore, the high signal observed in the T1-weighted image (T1WI) of the globus pallidus region, which has been utilized as a marker for ABE, may represent a transient phenomenon during the process of myelination [6]. The ABR has limited effectiveness in monitoring bilirubin-induced neurotoxicity beyond the auditory pathway [7]. As a crucial electrophysiological monitoring tool for evaluating neonatal brain function in the neonatal intensive care unit, amplitude-integrated electroencephalography (aEEG) has been extensively utilized in assessing bilirubin-induced brain injury due to its numerous advantages, including ease of operation and interpretation, non-invasive nature, real-time continuous bedside monitoring, and the capacity to detect abnormalities in the brain’s electrical activity that may be undetectable through imaging methods [8]. The bilirubin-induced neurological dysfunction (BIND) score serves as an objective tool for assessing the degree of neurological impairment and possesses high positive and negative predictive values in the diagnosis and grading of bilirubin encephalopathy [9, 10]. However, aEEG and BIND scores may be influenced by a variety of clinical and biochemical factors, and systematic analysis of these influencing factors remains insufficient. Therefore, this study conducted a retrospective analysis of 294 neonates with hyperbilirubinemia to investigate the potential influencing factors affecting the aEEG score and BIND score in the diagnosis of bilirubin-induced brain injury.

## Methods

### Participants

A total of 294 neonates diagnosed with hyperbilirubinemia and admitted to the Neonatology Department of Kunming Yan’an Hospital were included in this study. The inclusion criteria were: (1) gestational age ≥ 35 weeks; (2) serum bilirubin level exceeding the 95th percentile of the Bhutani curve [11]; (3) age at admission < 28 days; and (4) availability of complete clinical records. The exclusion criteria were: (1) the presence of neurological malformations or severe malformations in other systems; (2) the diagnosis of inherited metabolic disease;3) known hemolytic diseases, including Rh or ABO blood group incompatibility, glucose-6-phosphate dehydrogenase (G6PD) deficiency, or other hemolytic conditions; and 4) the complication of hypoglycemia, infection, intracranial hemorrhage, hypoxic-ischemic encephalopathy, or other disorders potentially causing brain damage. No clear cause was identified for the hyperbilirubinemia in the included subjects.

### Clinical data collection

Data were extracted from the electronic medical record system and included the following: (1) General information: gender, delivery mode, birth weight, birth length, maternal age, day age, gestational age, and corrected gestational age (CA). (2) Laboratory indices obtained at admission prior to any treatment: total bilirubin (, indirect bilirubin (IB), direct bilirubin (DB), albumin (ALB), TB/ALB (TB/A), IB/ALB (IB/A), and lactate dehydrogenase (LDH). (3) Cranial ultrasound findings: Abnormalities were defined as the presence of any abnormal finding on ultrasonography, including ventriculomegaly, periventricular/choroid plexus cysts, altered parenchymal echogenicity, subependymal abnormalities, or abnormal cerebral hemodynamics; neonates with reports indicating “no significant abnormalities detected” were classified as normal.

### The monitoring and scoring of aEEG

Bedside aEEG monitoring was performed within 48 h of admission using the Neonatal Electroencephalograph (VISHEE, CFM-I, Nanjing, China). Cerebral electrodes were placed according to the international 10/20 system, with the C3-P3 and C4-P4 double-lead montage selected, and the duration of recording was at least 3 h in each case. Based on the modified aEEG scoring method proposed by Xu et al. [12], the assessment was conducted by scoring three aspects of aEEG: continuity, sleep-wake cycle (SWC), and seizure activity (SA). The total score ranges from 3 to 12 points, with lower scores indicating more severe brain injury (details are provided in Supplementary File 1). Neonates with a perfect score were included in the normal aEEG score group, while the remaining neonates were included in the abnormal aEEG score group.

### Scoring of BIND

The BIND scores were assessed based on three aspects as described by Johnson et al. [13]: mental status, muscle tone, and crying, with each performance rated as 0 for normal, 1 for mild impairment, 2 for moderate impairment, and 3 for severe impairment. The total score for the three items was categorized as follows: 1–3 for mild abnormality, 4–6 for moderate abnormality, and 7–9 for severe abnormality. Neonates with a total BIND score of 0 were assigned to the normal BIND score group, while those with non-zero scores were categorized into the abnormal BIND score group.

### Standardization of assessments

Both aEEG and BIND assessments were performed by a single trained neonatologist on awake, spontaneously breathing infants without sedation. The assessor was blinded to laboratory results throughout scoring. For aEEG, artifact-free stable segments of ≥ 30 min were used for final analysis.

### Statistical analysis

Statistical analysis was conducted using SPSS version 26.0. Normal measurement data were expressed as mean ± standard deviation, while non-normal measurement data were presented as median (lower quartile, upper quartile), and categorical data were expressed as percentages.The independent samples t-test was employed for comparing normal measurement data between the two groups, while the Mann-Whitney U test was utilized for comparing non-normal measurement data between groups. Categorical data were compared using the chi-squared (χ²) test. Spearman’s correlation coefficient was applied to analyze the relationships between each index and both BIND score and aEEG score. Logistic regression analysis was performed to identify the influencing factors associated with abnormal BIND scores and abnormal aEEG scores, Variance inflation factors (VIFs) and tolerance were computed to assess multicollinearity, with VIF > 10 (tolerance < 0.1) defining significant collinearity. Receiver Operating Characteristic (ROC) curves and the area under the ROC curve (AUC) were utilized to evaluate the predictive value of each index for abnormal BIND scores and abnormal aEEG scores. A P value of less than 0.05 was considered statistically significant.

## Results

### Statistics on the number of neonates across each score band for both the BIND score and the aEEG score

Of the 294 neonates, 130 neonates (44.22%) were classified into the normal aEEG score group, while 164 neonates (55.78%) were categorized in the abnormal aEEG score group. The abnormal aEEG scores ranged from 7 to 11, with the highest number of neonates scoring 11 (97 cases) and only one neonate scoring 7. A total of 64 neonates (21.77%) were classified into the normal BIND score group, while 230 neonates (78.23%) were classified into the abnormal BIND score group, all of which exhibited mild abnormalities, with the highest proportion (68.37%) scoring 1 (Table 1).

**Table 1:** Statistics on the number of neonates across each score band for both the BIND score and the aEEG score

Group	Score	Number of cases	Percentage(%)
aEEG	7	1	0.34
	8	10	3.40
	9	12	4.08
	10	44	14.97
	11	97	32.99
	12	130	44.22
BIND	0	64	21.77
	1	201	68.37
	2	26	8.84
	3	3	1.02

All abnormal BIND scores were mild (scores 1–3), no moderate or severe cases (scores 4–9) were present in the subjects

### Univariate analysis of factors influencing abnormal BIND scores and abnormal aEEG scores

Compared to the abnormal BIND score group, the normal BIND score group exhibited lower levels of TB, DB, TB/A, and IB/A, as well as a higher level of LDH (all *P* < 0.05). No statistically significant differences were observed between the two groups concerning gender, delivery mode, birth weight, birth length, maternal age, gestational age, day age, CA, IB, ALB, and rates of cranial ultrasound abnormalities (*P* > 0.05).In comparison to the abnormal aEEG score group, the normal aEEG score group demonstrated lower levels of CA, TB, IB, TB/A, IB/A, and ALB (all *P* < 0.05). No significant differences were found between the two groups in terms of gender, birth weight, birth length, maternal age, gestational age, day age, delivery mode, DB, rates of cranial ultrasound abnormalities, and LDH (*P* > 0.05) (Table 2).

**Table 2:** Univariate analysis of factors influencing abnormal BIND scores and abnormal aEEG scores

Variables	BIND score group				aEEG score group			
	Normal	Abnormal	t/z/χ^2^	*P*	Normal	Abnormal	t/z/χ^2^	*P*
Male, n(%)	31(48.44%)	129(56.09%)	1.181	0.277	73(56.15%)	87(53.05%)	0.282	0.595
Female, n(%)	33(51.56%)	101(43.91%)			57(43.85%)	77(46.95%)		
Eutocia, n(%)	35(54.69%)	127(55.22%)	0.006	0.940	65(50.00%)	97(59.15%)	2.452	0.117
Caesarean section, n(%)	29(45.31%)	103(44.78%)			65(50.00%)	67(40.85%)		
Birth weight, g	3144 ± 421	3135 ± 469	0.136	0.892	3110 ± 417	3158 ± 489	-0.890	0.374
Birth length, cm	50.0(49.0,51.0)	50.0(25.0,75.0)	-0.357	0.721	50.0(49.0,51.0)	50.0(49.0,51.0)	-0.237	0.812
maternal age, y	29(27,34)	29(26,32)	-0.087	0.931	29(27,34)	29(26,32)	-1.005	0.315
Day age, d	5(3,7)	5(3,6)	-0.346	0.729	5(3,6)	5(3,6)	-0.896	0.370
Gestational age, w	38(38,39)	38(37,39)	-0.013	0.990	38(37,39)	38(38,39)	-1.923	0.055
CA, w	39(38,40)	39(38,40)	-0.537	0.592	39(38,39)	39(38,40)	-2.123	**0.034**
TB, umol/L	266.2(221.9,292.5)	282.8(228.7,346.0)	-2.499	**0.012**	251.8(219.5,286.0)	323.7(253.8,364.7)	-7.083	**< ** **0.001**
IB, umol/L	245.8(211.6,272.0)	257.0(205.9,313.4)	-1.835	0.067	224.2(197.3,256.3)	291.2(234.5,342.4)	-7.524	**< ** **0.001**
DB, umol/L	17.6(10.2,30.8)	23.5(14.6,34.0)	-2.083	**0.037**	23.5(13.9,33.5)	24.5(14.0,35.2)	-0.179	0.858
ALB, g/L	33.1(31.5,36.4)	33.2(31.0,36.4)	-0.725	0.469	32.7(30.9,35.5)	34.0(31.3,37.1)	-2.427	**0.015**
TB/A, umol/g	7.6 ± 2.0	8.6 ± 2.0	-3.312	**0.001**	7.6 ± 1.4	9.0 ± 2.1	-6.675	**<** ** 0.001**
IB/A, umol/g	7.1(6.3,8.4)	7.6(6.5,8.9)	-2.082	**0.037**	6.0(5.3,6.8)	7.0(5.8,8.3)	-6.816	**< ** **0.001**
Abnormal cranial ultrasound, n(%)	37(57.81%)	156(67.83%)	2.226	0.136	88(67.69%)	105(64.02%)	0.433	0.511
Normal cranial ultrasound, n(%)	27(42.19%)	74(32.17%)			42(32.31%)	59(35.98%)		
LDH, U/L	987(463,1305)	683(378,1132)	-2.696	**0.007**	868(442,1222)	683(369,1122)	-1.849	0.065

### Logistic regression analysis of influencing factors for abnormal BIND score and abnormal aEEG score

**Fig. 1:**
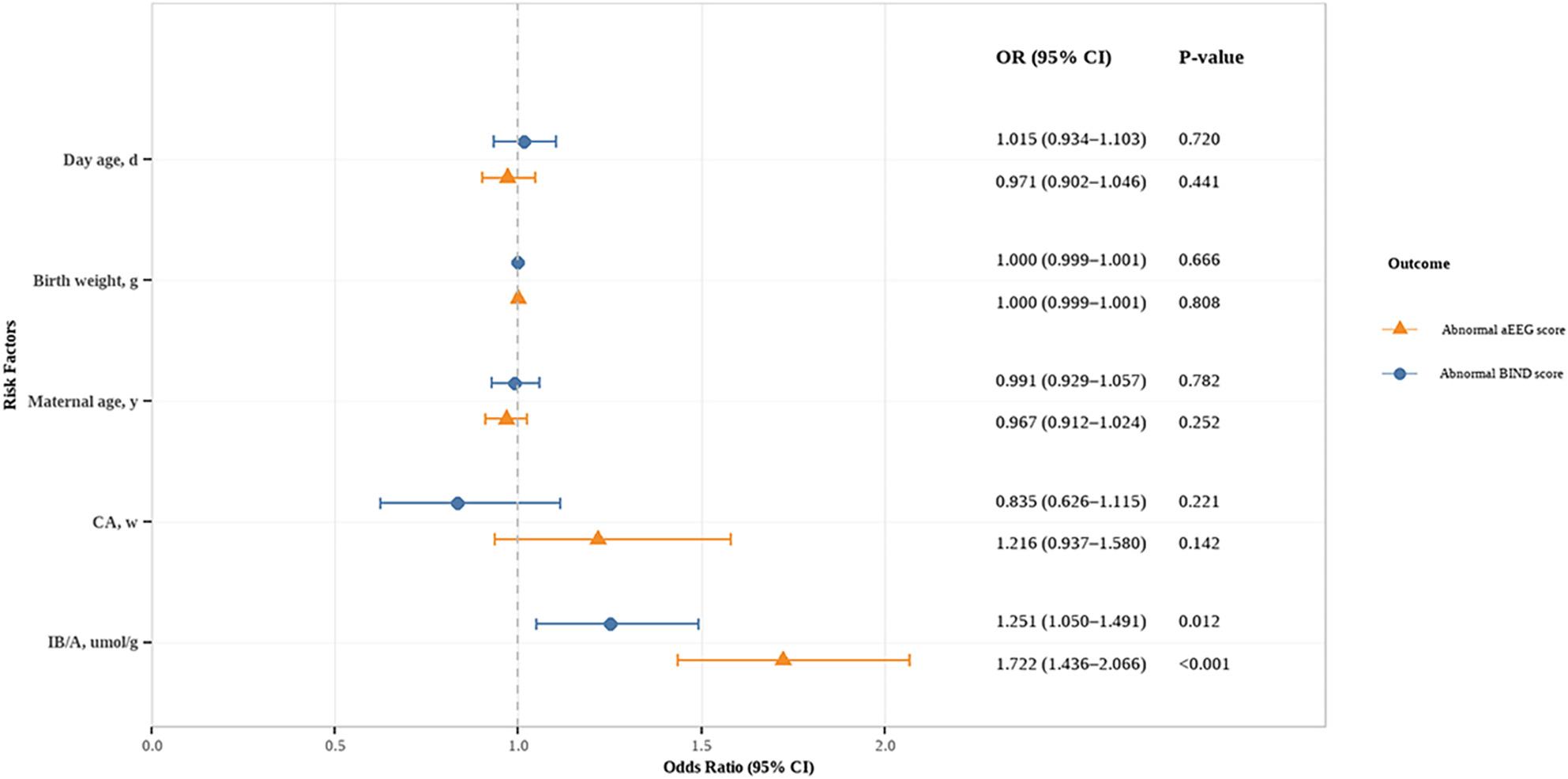
Forest plot of the multivariable logistic regression analysis for abnormal BIND and aEEG scores

Given that TB, IB, TB/A, and IB/A all reflect bilirubin levels and showed high collinearity (all VIF > 10.000, Supplementary File 2, Table 1), we selected IB/A, which can reflect the load state of free bilirubin with neurotoxicity [14], along with other variables such as day age, birth weight, maternal age, and CA, for inclusion in the multivariable logistic regression analysis. The results showed that IB/A was significantly associated with abnormal BIND scores (OR = 1.251, 95% CI = 1.050–1.491) and abnormal aEEG scores (OR = 1.722, 95% CI = 1.436–2.066), while no significant associations were observed between day age, birth weight, maternal age, or CA and either abnormal BIND or aEEG scores (all *P* > 0.05), as detailed in Fig. 1 and Supplementary File 2, Table 2. To assess the robustness of the primary model, we conducted sensitivity analyses by sequentially substituting TB/A, TB, and IB for IB/A, while keeping all other covariates constant. The results demonstrated that TB/A, TB, and IB remained positively associated with both outcomes (all *P* < 0.05), and the effect estimates for the other variables did not change materially (see Supplementary File 2, Table 3).

It is noteworthy that LDH, which showed a significant univariate association and has been reported in relation to bilirubin encephalopathy [15], was initially included in the multivariable logistic regression model for abnormal BIND scores (Supplementary File 2, Table 4). However, the inclusion of LDH rendered the association between IB/A and abnormal BIND scores statistically non-significant (OR = 1.182, *P* = 0.081). Moreover, the direction of the LDH effect (suggesting a protective association) lacked a plausible biological explanation. This pattern indicated potential residual confounding or model overfitting. Consequently, following the principle of model parsimony and to ensure the robustness and interpretability of the final model, LDH was excluded from the primary analysis reported herein.

### ROC curves for predicting abnormal BIND score and abnormal aEEG score using IB/A

**Fig. 2:**
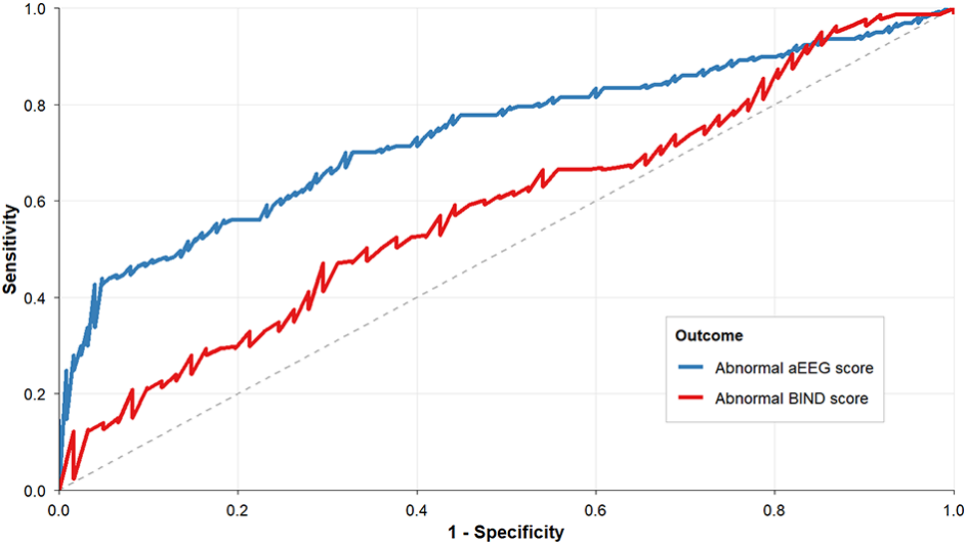
ROC curves for predicting abnormal BIND and aEEG scores using IB/A

An IB/A ratio greater than 7.7 identified abnormal BIND scores (AUC = 0.587, *P* < 0.05), while a ratio exceeding 8.7 identified abnormal aEEG scores (AUC = 0.736, *P* < 0.05). Both cut-offs demonstrated high specificity (70.5% and 95.2%, respectively) but low sensitivity (47.1% and 43.9%, respectively) (Fig. 2). Sensitivity analyses using the other three bilirubin-related indices yielded similar ROC results (Supplementary File 2, Table 5).

### Correlation analysis of BIND score, aEEG score and clinical data

**Fig. 3:**
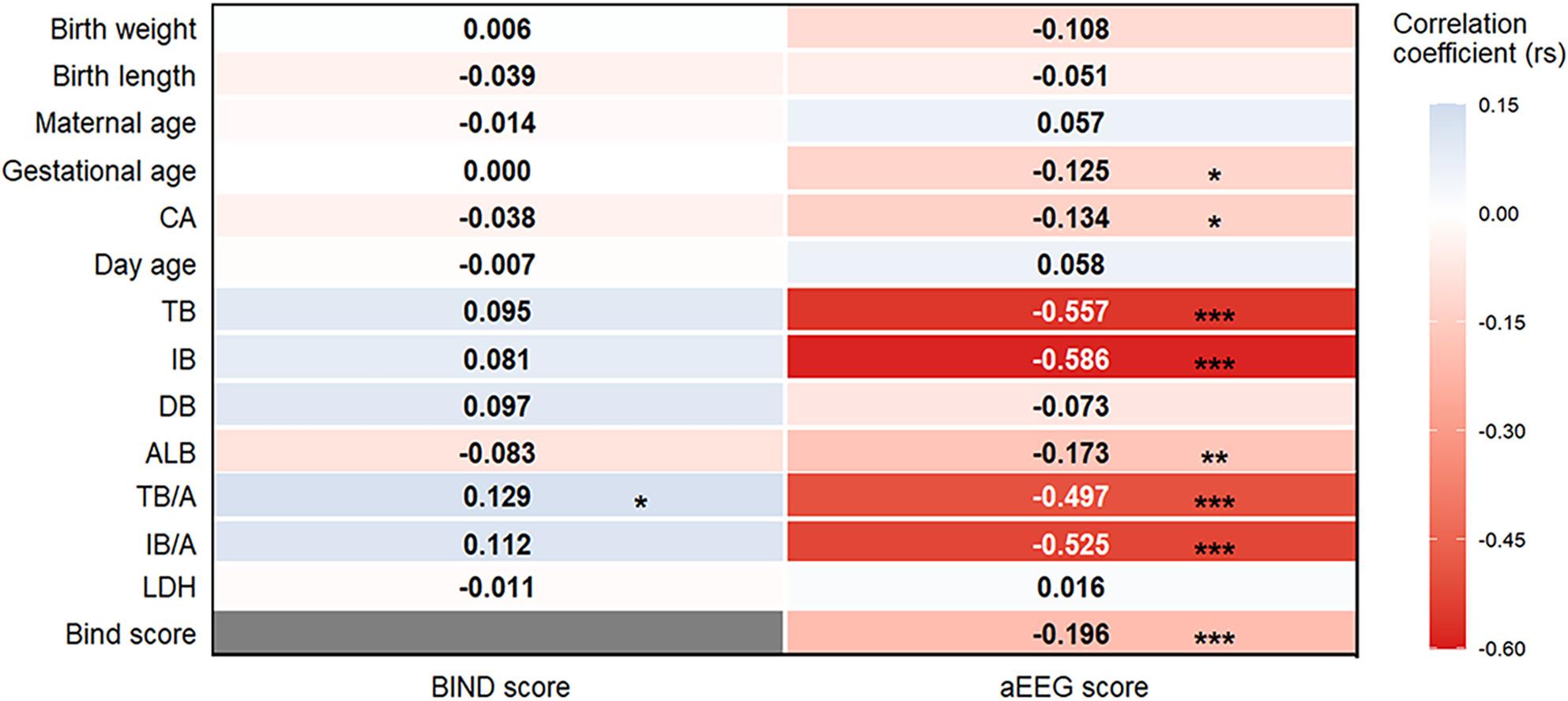
Correlation Analysis of BIND Score and aEEG Score with Clinical Data (*P < 0.05, **P < 0.01, ***P < 0.001)

The BIND score exhibited a positive correlation with TB/A (*P* < 0.05) and a negative correlation with the aEEG score (*P* < 0.001), whereas no significant linear associations were found between the BIND score and birth weight, birth length, maternal age, CA, day age, TB, IB, DB, IB/A, ALB, or LDH (all *P* > 0.05). The aEEG score was negatively correlated with TB, IB, TB/A, IB/A, ALB, and CA (all *P* < 0.05), but demonstrated no significant correlations with birth weight, birth length, maternal age, day age, DB, or LDH (all *P* > 0.05) (Fig. 3).

## Discussion

aEEG is a novel method for assessing neurological function, simplifying traditional EEG signals through amplification, compression, filtering, and other operations. It primarily evaluates background activity (including upper and lower boundary amplitude and continuity), SWC, and electrographic seizures in the recorded trace [16]. Several studies have demonstrated that the degree of aEEG abnormalities is positively correlated with the incidence of ABE, serving as a predictor of brain damage and a tool for assessing neurological complications in neonates with hyperbilirubinemia [8, 17–19]. The BIND score, which evaluates the severity of bilirubin-induced brain injury based on clinical manifestations, can effectively monitor the progression of ABE and predict poor prognoses [20]. In this study, a negative correlation was observed between the BIND score and the aEEG score, consistent with the findings reported by Chang [21]. This indicates that the two assessments exhibit substantial agreement regarding brain injury evaluation in neonates with hyperbilirubinemia and can serve as complementary tools for cross-validation in clinical practice.

The occurrence of bilirubin-induced brain injury is associated with the accumulation of high serum levels of UB across the blood-brain barrier in specific regions, including the globus pallidus, subthalamic nucleus, brainstem, substantia nigra, and cerebellum. This accumulation leads to oxidative stress, inflammation, gene expression imbalance, apoptosis, and necrosis, ultimately resulting in damage to the central nervous system [22, 23]. Although aEEG signals originate from the cerebral cortex, their background activity is closely associated with the electrical activity of deeper brain structures, such as the corticobasal ganglia-thalamic loop. Disruption of this loop by bilirubin may lead to abnormalities in the amplitude, frequency, and continuity of the electroencephalogram [8, 24]. The results of this study demonstrate that serum TB and IB levels are negatively correlated with aEEG scores and are associated factors for abnormal aEEG and BIND scores. This overall trend aligns with previous reports on the influence of bilirubin load on electroencephalographic activity. For example, Gürses et al. [25] observed a reduction in EEG amplitude with increasing bilirubin levels. Concurrently, the density, duration, and frequency of sleep spindles were significantly decreased in infants with hyperbilirubinemia, while the rate of asynchrony increased [26]. Zhang et al. [24] reported a reduction or even disappearance of SWC with rising TB levels. However, some studies have presented different conclusions. For instance, Ter Horst et al. [27], in a study of preterm infants with gestational ages between 26 and 31 weeks, found no synchronous direct effect of TB on aEEG amplitude. The researchers suggested this might be related to lower bilirubin exposure levels, but they observed a delayed effect on electroencephalographic activity during the second week after birth. These discrepancies suggest that the impact of bilirubin on electroencephalographic activity may be modulated by various factors, including exposure levels, gestational age, and timing of assessment. This study primarily included term and late-preterm infantss, all of whom had bilirubin levels exceeding the phototherapy intervention threshold. The observed association between bilirubin levels and abnormalities in aEEG and BIND scores provides further clinical data supporting the influence of bilirubin load on bedside neurofunctional assessments within this specific population.

The neurotoxicity of bilirubin is closely linked to its unbound state. ALB binds to UB, restricting its passage across the blood–brain barrier [28]. Therefore, in clinical practice where direct measurement of free bilirubin is not widely available, TB/A and IB/A have become strong indirect indicators for assessing the risk of bilirubin neurotoxicity [29, 30]. Particularly in high-risk neonates, when DB is elevated (≥ 2 mg/dL or DB/TB ≥ 20%), conventionally measured UB values may be artifactually high. In such cases, using IB/A to estimate the level of free bilirubin can provide a more reliable basis for risk assessment [31]. Based on this rationale, this study selected IB/A, which theoretically more directly reflects neurotoxic free bilirubin load and is less affected by DB interference [14], as a core variable. We found that IB/A showed a significant association with both abnormal aEEG scores and abnormal BIND scores. Sensitivity analysis further indicated that TB/A exhibited a similar association with abnormalities in both scores, supporting the link between B/A and bedside neurofunctional abnormalities. However, our findings also highlight important limitations in the clinical application of B/A. Although statistically significant, TB/A and IB/A demonstrated limited ability to discriminate abnormal BIND scores (AUC only 0.587–0.608), with cutoff values characterized by high specificity but low sensitivity. A similar pattern was observed for identifying abnormal aEEG scores. This suggests that in a population with predominantly mild neurological dysfunction, using B/A alone, while useful as a marker of abnormal bedside assessments, lacks sufficient sensitivity and may lead to underdiagnosis, consistent with the findings of Chang et al. [21]. Several factors may explain these observations. First, aEEG and BIND scores may be influenced by various non-bilirubin factors, including brain maturity and perinatal complications [32–36]. Second, ALB, as a dynamic carrier in bilirubin metabolism, has a binding capacity that is affected by the internal milieu (e.g., acidosis, infection) [37, 38], and its serum level may be more indicative of the body’s synthetic function or stress state than its real-time bilirubin-binding efficacy. Thus, the negative correlation between ALB levels and aEEG scores observed here may reflect this complex dissociation between ALB concentration and its functional efficacy.

In this study, CA showed a weak negative correlation with aEEG scores, which appears inconsistent with the conventional view that electroencephalographic activity becomes more organized with advancing brain maturation [39, 40]. However, in the multifactorial logistic regression models that incorporated bilirubin indices, CA did not demonstrate a statistically significant association with abnormalities in either aEEG or BIND scores. One possible explanation is that in this predominantly term and late-preterm infants population, the inhibitory effect of significant bilirubin neurotoxicity on background electroencephalographic activity may partially mask the positive influence of increasing age on EEG maturation. No significant effect of gestational age on BIND scores was observed in our study. A study by Liu et al. [41] involving preterm infants with gestational ages of 25.3–36.6 weeks and birth weights of 800–2300 g identified birth weight < 1.5 kg as a significant risk factor for abnormal aEEG. In comparison, our study, which included infants with gestational ages ≥ 35 weeks and birth weights > 2.00 kg, did not find a correlation between birth weight and either aEEG or BIND scores. This finding is consistent with results reported by Xu et al. [42] in preterm infants of 26–32 weeks’ gestation, suggesting that the impact of birth weight on aEEG may differ across gestational age and weight ranges. LDH, a sensitive marker of tissue injury, has been reported to correlate with the severity of bilirubin encephalopathy [15, 43] and can be elevated in models of bilirubin neurotoxicity [44]. In this study, however, LDH levels were higher in the group with normal BIND scores than in the abnormal group. Nevertheless, after adjusting for bilirubin load and other variables in the multifactorial model, the association between LDH and abnormal BIND scores lost statistical significance. Moreover, correlation analysis did not reveal any significant relationship between LDH and either score. This suggests that in our study population, variations in LDH may primarily reflect underlying and incompletely measured confounding factors, such as unrecognized perinatal stress, acidosis, or other conditions known to markedly elevate LDH [45, 46]. This highlights the necessity of considering a broad range of confounders when interpreting nonspecific biomarkers. Additionally, factors such as birth length, maternal age, delivery mode, and day age did not show any association with abnormalities in aEEG or BIND scores within this study population.

This study has several limitations. First, the retrospective, cross‑sectional, and single‑center design is inherently exploratory and can demonstrate associations but does not establish causality or provide robust predictive validity. The findings should be considered hypothesis‑generating and require prospective validation. Second, the sample size was limited, which may have constrained our ability to detect significant associations, particularly in subgroup analyses. Future multi‑center studies with larger cohorts are needed. Third, the cohort primarily consisted of infants with mild abnormalities (BIND scores 1–3), limiting the generalizability of findings to cases of moderate or severe bilirubin encephalopathy. Fourth, while we used standardized tools, the aEEG and BIND scores involve a degree of clinical subjectivity. Objective neuroimaging (e.g., cranial MRI) or electrophysiological (e.g., ABR) correlates were not routinely available for validation in this clinical setting.

In summary, this study of neonates with hyperbilirubinemia at gestational age ≥ 35 weeks and predominantly mild neurological abnormalities revealed that IB/A is an independent core factor influencing abnormal aEEG and BIND scores. The association trends were consistent with those of TB/A, TB, and IB, confirming the key regulatory role of serum bilirubin load on bedside neurofunctional assessment outcomes. The aEEG and BIND scores demonstrated good consistency in reflecting bilirubin neurotoxicity, offering a complementary and mutually verifiable bedside assessment strategy for clinical practice. Conventional demographic and perinatal indicators such as birth weight, birth length, maternal age, delivery mode, and day age did not show significant effects on the two scores in this study population, suggesting that these factors may not be critical confounders in assessing bilirubin neurotoxicity in this specific group. It is important to note that bilirubin-related indices exhibited high specificity but low sensitivity in identifying aEEG and BIND abnormalities, indicating that their isolated application may lead to underdiagnosis. Clinically, integrating multidimensional indicators is essential to improve diagnostic accuracy. This study provides clinical evidence supporting the use of bedside tools for early assessment of bilirubin neurotoxicity risk and offers a reference for further clarifying the predictive value and application boundaries of aEEG and BIND scores in future research.

## Supplementary Information

Supplementary Material 1.

Supplementary Material 2.

## Data Availability

The datasets used and/or analysed during the current study are available from the corresponding author on reasonable request.

## Abbreviations

ABE: Acute bilirubin encephalopathy
ABR: Auditory brainstem response
aEEG: Amplitude-integrated electroencephalography
ALB: Albumin
AUC: Area under the receiver operating characteristic curve
BIND: Bilirubin-induced neurological dysfunction
CA: Corrected gestational age
CI: Confidence interval
DB: Direct bilirubin
IB: Indirect bilirubin
IB/A: Indirect bilirubin / albumin
LDH: Lactate dehydrogenase
MRI: Magnetic resonance imaging
OR: Odds ratio
ROC: Receiver operating characteristic
SWC: Sleep-wake cycling
T1WI: T1-weighted image
TB: Total bilirubin
TB/A: Total bilirubin / albumin
UB: Unconjugated bilirubin
