# Supplementary material for: The influencing factors of amplitude-integrated electroencephalography and bilirubin-induced neurological dysfunction scores in neonates with hyperbilirubinemia: a cross-sectional study
Source: BMC Pediatr. 2026 Mar 6;26:307. doi: 10.1186/s12887-026-06700-1 (PMC13078049; doi:10.1186/s12887-026-06700-1)
Supplement: Supplementary file 1 — Supplementary Material 1. [file 12887_2026_6700_MOESM1_ESM.pdf]

Supplementary Table 1. Multicollinearity diagnostics among bilirubin-related indicators

| Factor | Abnormal BIND score | | Abnormal aEEG score | |
|---|---|---|---|---|
| | Tolerance | VIF | Tolerance | VIF |
| TB, umol/L | 0.001 | 1264.930 | 0.001 | 1264.930 |
| IB, umol/L | 0.001 | 1404.598 | 0.001 | 1404.598 |
| TB/A, umol/g | 0.001 | 1118.315 | 0.001 | 1118.315 |
| IB/A, umol/g | 0.001 | 1254.626 | 0.001 | 1254.626 |

Supplementary Table 2. Multivariable logistic regression analysis for abnormal BIND score and abnormal aEEG score.

| Outcome | Factor | B | SE | Wald | OR | 95%CI | *P* | Tolerance | VIF |
|---|---|---|---|---|---|---|---|---|---|
| Abnormal BIND score | Day age, d | 0.015 | 0.042 | 0.128 | 1.015 | 0.934-1.103 | 0.720 | 0.881 | 1.135 |
| | Birth weight, g | <0.001 | 0.000 | 0.186 | 1.000 | 0.999-1.001 | 0.666 | 0.907 | 1.102 |
| | Maternal age, y | -0.009 | 0.033 | 0.077 | 0.991 | 0.929-1.057 | 0.782 | 0.978 | 1.022 |
| | CA, w | -0.180 | 0.147 | 1.495 | 0.835 | 0.626-1.115 | 0.221 | 0.865 | 1.155 |
| | IB/A, umol/g | 0.224 | 0.089 | 6.286 | 1.251 | 1.050-1.491 | **0.012** | 0.943 | 1.061 |
| Abnormal aEEG score | Day age, d | -0.029 | 0.038 | 0.595 | 0.971 | 0.902-1.046 | 0.441 | 0.881 | 1.135 |
| | Birth weight, g | 0.000 | 0.000 | 0.059 | 1.000 | 0.999-1.001 | 0.808 | 0.907 | 1.102 |
| | Maternal age, y | -0.034 | 0.030 | 1.313 | 0.967 | 0.912-1.024 | 0.252 | 0.978 | 1.022 |
| | CA, w | 0.196 | 0.133 | 2.156 | 1.216 | 0.937-1.580 | 0.142 | 0.865 | 1.155 |
| | IB/A, umol/g | 0.544 | 0.093 | 34.335 | 1.722 | 1.436-2.066 | **<0.001** | 0.943 | 1.061 |

Supplementary Table 3. Multivariable logistic regression analysis using different bilirubin indicators.

| Model | Abnormal BIND score | | Abnormal aEEG score | |
|---|---|---|---|---|
| | OR (95%CI) | *P* | OR (95%CI) | *P* |
| Alternative Model 1: TB, umol/L | 1.007(1.002-1.011) | 0.005 | 1.015(1.010-1.020) | **<0.001** |
| Alternative Model 2: IB, umol/L | 1.006(1.001-1.011) | 0.019 | 1.018(1.013-1.023) | **<0.001** |
| Alternative Model 3: TB/A, umol/g | 1.293(1.097-1.525) | 0.002 | 1.568(1.341-1.834) | **<0.001** |

Note: All models were adjusted for the same set of covariates as the primary model (Day age, Birth weight, Maternal age, CA). Multicollinearity was assessed and confirmed to be absent among variables within each respective model.

Supplementary Table 4. Multivariable logistic regression model for abnormal BIND score including LDH.

| Factor | B | SE | Wald | OR | 95%CI | *P* | Tolerance | VIF |
|---|---|---|---|---|---|---|---|---|
| Day age, d | -0.021 | 0.044 | 0.236 | 0.979 | 0.898-1.067 | 0.627 | 0.839 | 1.191 |
| Birth weight, g | 0.000 | 0.000 | 0.139 | 1.000 | 0.999-1.001 | 0.710 | 0.896 | 1.116 |
| Maternal age, y | -0.009 | 0.036 | 0.069 | 0.991 | 0.923-1.063 | 0.793 | 0.960 | 1.042 |
| CA, w | -0.145 | 0.164 | 0.783 | 0.865 | 0.627-1.193 | 0.376 | 0.825 | 1.213 |
| IB/A, umol/g | 0.168 | 0.096 | 3.045 | 1.182 | 0.980-1.427 | 0.081 | 0.916 | 1.092 |
| LDH, U/L | -0.001 | 0.000 | 4.433 | 0.999 | 0.999-1.000 | **0.035** | 0.962 | 1.040 |

Supplementary Table 5. Results of ROC analysis for predicting abnormal BIND score and abnormal aEEG score using different bilirubin indicators.

| Outcome | Factor | AUC | 95% CI Lower bound | 95% CI Upper bound | *P* | Cutoff value | Sensitivity | Specificity |
|---|---|---|---|---|---|---|---|---|
| Abnormal BIND score | TB, umol/L | 0.602 | 0.528 | 0.676 | **0.012** | 303.6 | 0.422 | 0.813 |
| | IB, umol/L | 0.577 | 0.501 | 0.652 | 0.067 | 290.2 | 0.353 | 0.869 |
| | TB/A, umol/g | 0.608 | 0.531 | 0.684 | **0.008** | 8.7 | 0.443 | 0.750 |
| | IB/A, umol/g | 0.587 | 0.509 | 0.666 | **0.037** | 7.7 | 0.471 | 0.705 |
| Abnormal aEEG score | TB, umol/L | 0.741 | 0.685 | 0.797 | **<0.001** | 314.0 | 0.500 | 0.938 |
| | IB, umol/L | 0.761 | 0.706 | 0.816 | **<0.001** | 293.0 | 0.484 | 0.944 |
| | TB/A, umol/g | 0.714 | 0.656 | 0.772 | **<0.001** | 9.2 | 0.482 | 0.900 |
| | IB/A, umol/g | 0.736 | 0.679 | 0.794 | **<0.001** | 8.7 | 0.439 | 0.952 |
