# Supplementary material for: The influencing factors of amplitude-integrated electroencephalography and bilirubin-induced neurological dysfunction scores in neonates with hyperbilirubinemia: a cross-sectional study
Source: BMC Pediatr. 2026 Mar 6;26:307. doi: 10.1186/s12887-026-06700-1 (PMC13078049; doi:10.1186/s12887-026-06700-1)
Supplement: Supplementary file 2 — Supplementary Material 2. [file 12887_2026_6700_MOESM2_ESM.pdf]

Modified aEEG Scoring Criteria

Score	Continuity	SWC	SA
1	Flat trace (FT)	No sleep-wake cycle	Status epilepticus: continuous epileptiform discharges lasting >30 min
2	Continuous low voltage (CLV)	Immature sleep-wake cycle	Repetitive seizures: >1 episode within 30 min
3	Burst suppression (BS)	Mature sleep-wake cycle	Single seizure: isolated epileptiform discharge
4	Discontinuous normal voltage (DNV)	-	No seizures
5	Continuous normal voltage (CNV)	-	-

Detailed Scoring Standards for Each Analytical Parameter:

1. Continuity

Score	Continuity Type	Definition	Voltage Criteria
5	Continuous normal voltage (CNV)	Continuous electrical activity with regular bandwidth and no significant amplitude variation	Upper margin >10 μV, lower margin >5 μV
4	Discontinuous normal voltage (DNV)	Discontinuous electrical activity with irregular bandwidth and significant amplitude variation	Upper margin >10 μV, lower margin ≤5 μV
3	Burst suppression (BS)	Discontinuous electrical activity with sustained very low voltage in the lower margin (0–2 μV) interspersed with high-amplitude bursts in the upper margin	Lower margin: 0–2 μV<br>Burst voltage: >25 μV
2	Continuous low voltage (CLV)	Continuous very low electrical activity	Upper margin <10 μV
1	Flat trace (FT)	Isoelectric line activity, equivalent to electrocerebral silence	Upper margin <5 μV

2. Sleep-Wake Cycle (SWC)

Score	SWC Type	Definition	Duration/Characteristics
3	Mature sleep-wake cycle	Clear and distinct sinusoidal-like variation (smooth sinusoidal curve changes, mainly in the lower margin)	Cycle duration >20 min
2	Immature sleep-wake cycle	Some cycles present but unclear or interrupted	-
1	No sleep-wake cycle	Absence of sinusoidal-like variation	-

3. Seizure Activity (SA)

Score	SA Type	Definition	Duration/Frequency
1	Status epilepticus	Continuous epileptiform discharges	Lasting >30 min
2	Repetitive seizures	More than one episode within 30 min	-
3	Single seizure	Isolated epileptiform discharge	-
4	No seizures	Absence of epileptiform discharges	-
